# Tuning Magnetic Nanoparticles: Effect of Temperature on the Formation of Magnetite and Hematite Phases

**DOI:** 10.1021/acsomega.5c02750

**Published:** 2025-06-11

**Authors:** Frederico Vieira Gutierrez, Sonia Letichevsky, Anna De Falco, Beatriz Marques Ereias, Caique Diego de Abreu Lima, Wanessa Afonso de Andrade, Lanna Isabely Morais Sinimbu, Thais Cristina Viana de Carvalho, Bruno Gomes Silva, Rubem Luis Sommer, Geronimo Perez, Liying Liu, Jefferson Ferraz Damasceno Felix Araujo

**Affiliations:** † Department of Physics, 28099 Pontifical Catholic University of Rio de Janeiro, Rua Marques de São Vicente, 225, Rio de Janeiro 22451-900, Brazil; ‡ Department of Chemical and Materials Engineering, 28099 Pontifical Catholic University of Rio de Janeiro, Rua Marques de São Vicente, 225, Rio de Janeiro 22451-900, Brazil; § New Wave Tech, Av. Mascarenhas de Morais, 2231, Rio de Janeiro 25230-030, Brazil; ∥ Center for Biotechnology and Interdisciplinary Sciences, 8024 Rensselaer Polytechnic Institute, Troy, New York 12180, United States; ⊥ Department of Chemistry and Chemical Biology, 8024 Rensselaer Polytechnic Institute, Troy, New York 12180, United States; # NIT − National Institute of Technology, Venezuela Avenue, 82, Rio de Janeiro 20081-312, Brazil; ∇ Brazilian Center for Research in Physics, Rio de Janeiro 22290-180, Brazil; ○ Department of Mechanical Engineering, Universidade Federal Fluminense, Niteroi 24210-240, Brazil

## Abstract

Magnetic nanoparticles up to 15 nm, such as magnetite (Fe_3_O_4_), exhibit superparamagnetic properties at room temperature. These nanoparticles are being explored for medical applications, and the coprecipitation method is favored for its cost-effective production and scalability. This study investigates the impact of synthesis temperature on the nanoparticles’ physicochemical characteristics. The samples were synthesized at three different temperatures: 60, 70, and 80 °C (T60, T70 and T80). Analysis carried out using X-ray diffraction and Raman spectroscopy techniques revealed that the sample T60 is magnetite. As the temperature increased, the presence of hematite (Fe_2_O_3_) was detected in samples T70 and T80. In % mass, it was 8.0% for T70 and 8.7% for T80. Raman spectroscopy showed the characteristic bands of the magnetite phase at 341, 500, and 680 cm^–1^ and a low percentage of hematite present in the samples T70 and T80. The presence of hematite in the samples offers several advantages, including enhanced catalytic and magnetic properties, improved adsorption of contaminants, and greater thermal and chemical stability. Through various characterization techniques, including XRD, Raman spectroscopy, and transmission electron microscopy (TEM), the average diameter of the magnetic nanoparticles was confirmed to be approximately 5 nm. The coprecipitation route proved efficient for producing magnetite nanoparticles at temperatures below 70 °C. For specific applications, synthesis at temperatures above 70 °C may yield nanoparticles with a small proportion of hematite, introducing new functional properties that broaden their potential applications, such as in catalysis or environmental remediation.

## Graphical abstract

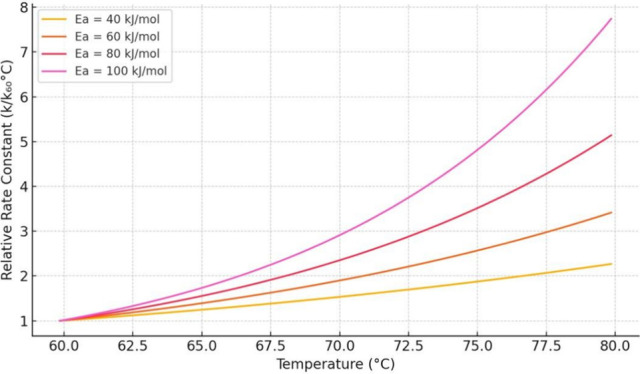

## 1 Introduction

Magnetite, widely studied and explored by the scientific community, is known for its remarkable magnetic properties. Its key characteristic is its ability to be attracted to magnets and to act as a permanent magnet when magnetized. This magnetic behavior arises from the unique crystalline structure of the cubic spinel phase, composed of ferrous ions (Fe^2+^) and ferric ions (Fe^3+^). The arrangement of these ions in the spinel structure creates a strong internal magnetic field, giving magnetite its distinctive ferromagnetic properties.^1–5^

Particularly in the nanoscale, below approximately 15 nm, magnetite presents superparamagnetic properties. These properties have attracted significant interest in the scientific world due to their advantages, such as the absence of hysteresis, avoiding residual magnetization after removal of the magnetic field, and high magnetic sensitivity, allowing precise control in applications such as magnetic separation and targeting. Its colloidal stability reduces the formation of agglomerates, favoring its use in liquid solutions and biological systems. These particles also stand out in biomedicine, being widely used in imaging diagnosis, hyperthermia therapy and drug delivery systems due to their biocompatibility and chemical functionalization capacity. Furthermore, their size is controlled during synthesis to optimize properties and have less energy dissipation through heat, making them ideal for efficient and sustainable magnetic devices.^1–4,6^ This particular set of properties is notably important since magnetic nanoparticles (MNPs) demonstrate a lack of remanence upon application of a magnetic field. The particles in question undergo a total loss of their magnetic properties, a process known as complete demagnetization. This phenomenon distinguishes them from bulk magnetite, as well as from other ferromagnetic and ferrimagnetic materials that retain their magnetization under certain conditions.^7^ Several methods are used to produce magnetite nanoparticles, including hydrothermal,^7^ sol–gel,^8^ microemulsions,^9^ chemical vapor deposition (CVD),^10^ coprecipitation,^6^ etc. The synthesis route significantly impacts the quantity of sample produced, the precision of nanoparticle size control, the morphology of the particles, the extent of agglomeration, and the purity of the material.^11^

This research applied the coprecipitation method because of its effectiveness already reported by Gutierrez et al.^4^ In addition to being simple and rapid, this method provides high yields, purity, and low costs, resulting in a significant quantity of nanoparticles with minimal oxidation. The material integrity over an extended duration is verified, and the method is easily scalable. It provides highly stable nanoparticles with high biocompatibility.^2,4,5,11^

The coprecipitation method described by Arsalani represents an innovative approach for producing functionalized nanoparticles. This technique is particularly valuable as it involves applying temperature to the precipitating agent, which is an NH_4_OH solution. This method enhances the efficiency and effectiveness of nanoparticle synthesis.^1^ In addition, this method offers precise size control without affecting the material’s magnetic behavior.^1,5^ Using this technique, Gutierrez showed that it is possible to maintain the integrity of the magnetite core by varying the temperature of the ammonia-precipitating agent.^4^ The results obtained indicate a possibility of reducing energy and time in the production of magnetic nanoparticles (MNPs) compared to the parameters used in Arsalani.^1^ However, at elevated temperatures, the nanoparticles may become destabilized during nucleation, leading to the formation of other iron oxides, such as hematite (Fe_2_O_3_).

Hematite is well-known for its reddish color, trigonal or rhombohedral crystalline structure, and for its semiconductor properties; in addition to being antiferromagnetic at room temperature, it can exhibit ferromagnetic behavior under certain conditions.^12^ The presence of hematite in magnetite samples offers advantages that broaden their applications. Hematite acts as a catalyst in oxidation reactions.^13,14^ The combination of Fe_2_O_3_ and Fe_3_O_4_ benefits catalytic processes by facilitating redox cycle.^13^ For environmental applications, the presence of hematite can increase the adsorption capacity of certain contaminants in aqueous solutions.^15^ Hematite is known to enhance the chemical and physical stability of nanoparticles. Its presence can help prevent the degradation of magnetite, particularly in conditions and applications that involve elevated temperatures. This stability is essential for maintaining the effectiveness and longevity of nanoparticles in various usage scenarios.^13^ It can reduce the aggregation tendency of magnetite nanoparticles.^13^ For applications in biomedicine, hematite has contrast properties, which can enable the application of magnetite nanoparticles to provide adjustable contrast in images and enhance the thermal response of nanoparticles, improving the efficiency of hyperthermia treatment.^16^

In this work, various analytical techniques were used to characterize the physical and magnetic properties of the nanoparticle samples, including their crystallinity, morphology, size, distribution, and overall behavior. Structural analysis confirmed the presence of magnetite as the primary phase, with variations in composition observed at different synthesis temperatures. Magnetic properties were assessed using specialized measurement equipment. Overall, the findings highlight that adjusting the synthesis temperature, magnetic nanoparticles with tunable properties can be produced. The MNPs are suitable for a wide range of applications, including biomedical and imaging technologies.

## 2 Experimental Procedure

The MNPs were prepared by the coprecipitation method using ferric chloride hexahydrate (FeCl_3_·6H_2_O) (Vetec); iron II sulfate heptahydrate (FeSO_4_·7H_2_O), (Vetec); hydrochloric acid (HCl) (Isofar); ammonium hydroxide (NH_4_OH, 28%) (SIGMA) and distilled water.

Two separate solutions containing Fe^3+^ and Fe^2+^ ions are necessary to produce magnetite samples. For the Fe^3+^ solution, the reagent FeCl_3_·6H_2_O was diluted in distilled water, reaching a concentration of 1.0 mol L^–1^ (eq 1). In another beaker, FeSO_4_·7H_2_O was dissolved in an HCl solution with a concentration of 5.49 mol L^–1^, resulting in a concentration of 0.62 mol L^–1^ (eq 2), forming the Fe^2+^ solution, for the formation of magnetite nanoparticles.^4^

$${\mathrm{FeCl}}_{3}\cdot 6H_{2}O_{\left(s\right)}\to {\mathrm{Fe}}^{3+}+3{\mathrm{Cl}}^{-}+6H_{2}O$$ 1

$${\mathrm{FeSO}}_{4}\cdot 7H_{2}O_{\left(s\right)}+2\mathrm{HCl}\to {\mathrm{Fe}}^{2+}+{{\mathrm{SO}}_{4}}^{2-}+7H_{2}O+2H^{+}+2{\mathrm{Cl}}^{-}$$ 2

The two iron solutions were mixed in a 2:1 ratio of Fe^3+^ to Fe^2+^, forming a homogeneous iron salt solution. In a third beaker, a solution of NH_4_OH (1.3 mol L^–1^) was prepared and placed on a heating plate until the synthesis temperature was achieved (60, 70, and 80 °C). After the ammonia solution reached the desired temperature, the solution was kept for 10 min for stabilization. The homogeneous solution of iron salts was added dropwise with a pipet to the ammonia solution under constant stirring. As soon as the iron salt solution reacts with the basic solution (eq 3), it is possible to see the black precipitate formation instantly.^17,18^

$$2{\mathrm{Fe}}^{3+}+{\mathrm{Fe}}^{2+}+8{\mathrm{OH}}^{-}\to {\mathrm{Fe}}_{3}O_{4\left(s\right)}+4H_{2}O$$ 3

Once the coprecipitation process is complete, all samples are placed in an ultrasound bath for 1 h to prevent particles agglomeration. Finally, the samples are washed at least three times with distilled water to neutralize the solution’s pH.

For this work, the temperatures explored were 60, 70, and 80 °C, and the samples were named T60, T70, and T80, respectively. All samples were produced in the Biophysics Laboratory and Materials Treatment of the Physics Department of the Pontifical Catholic University of Rio de Janeiro—RJ.

## 3 Characterization Techniques

### 3.1 X-ray Diffraction (XRD)

The XRD analysis was performed in a Bruker D8 Discover diffractometer equipped with a copper tube operating at 40 mA and 40 kV, a nickel filter and a Lynxeye detector. The analyses were obtained in the 2 range 20°–90°, 15 s and 0.02° step. The results were analyzed using the Rietveld refinement method using the fundamental parameters approach in the program Topas 5.0.

### 3.2 Raman Spectroscopy

Raman measurements were performed at room temperature using a LabRAM HR Evolution Raman spectrometer (HORIBA) equipped with an Olympus microscope and an x100_VIS_LWD objective. A 638 nm laser source was employed to generate the Raman spectra, with the focus precisely adjusted on the sample surface to attain a resolution of 4 cm^–1^. The spectra were collected through 2 accumulations, each lasting 40 s.

### 3.3 Transmission Electron Microscopy

The morphology was examined using field-emission transmission electron microscopy (TEM, JEOL JEM 2100F/JEOL) in both TEM and scanning-TEM (STEM) modes at 200 kV. Elemental mapping was conducted using Energy-Dispersive X-ray Spectroscopy (EDS) in STEM mode. For sample preparation, isopropanol suspensions of the materials were drop-cast onto carbon-coated copper grids and allowed to air-dry at room temperature.

### 3.4 Electrical Measurements

The four-point probe method was implemented using a Keithley SourceMeter (Model 2401). Samples were mounted in a measurement setup with copper probes acting as electrical contacts for the magnetic particles. The configuration consisted of a Cu/Sample/Cu structure, with an electrode contact area of 0.58 ± 0.09 cm^2^. The average sample thickness was 0.50 ± 0.08 mm.

### 3.5 Reflectance

The studied samples’ reflectance was measured using the PerkinElmer Lambda 950 UV–vis–NIR Spectrophotometer, which has two lamps: one deuterium (UV) and the other tungsten-halogen (VIS–NIR). The integrating sphere module was used to measure the absolute reflectance in the visible region.

### 3.6 Magnetic Measurements

Magnetization measurements were performed using the VSM module of the Quantum Design PPMS DynaCool system to analyze the samples’ magnetic properties. An external DC magnetic field was applied in the range of ±20 kOe to obtain the magnetization curves. Additionally, magnetic measurements were conducted across a temperature range of 2–400 K to explore the samples’ temperature-dependent magnetic behavior.

## 4 Results and Discussion

### 4.1 X-ray Diffraction

Figure 1 presents the XRD patterns of the synthesized samples T60 (black curve), T70 (red curve), and T80 (blue curve). All samples exhibit the characteristic peaks of magnetite, Fe_3_O_4_, at 30°, 35°, 43°, 54°, 57°, and 63°, corresponding to the (220), (311), (400), (422), (511), and (440) planes, respectively.

**Figure 1:**
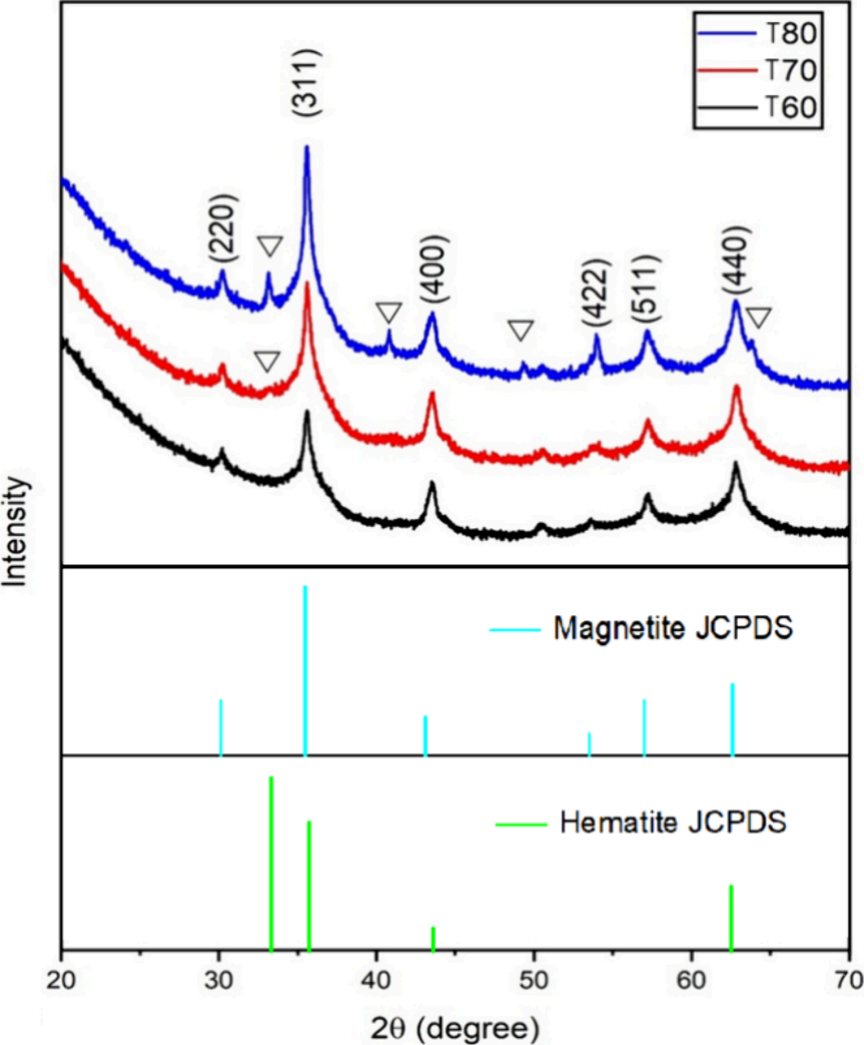
XRD patterns of samples T60, T70, and T80. The peaks marked with the inverted triangle (∇) represent the hematite phase observed in the T70 and T80 samples.

XRD analysis reveals that the magnetite diffraction peaks exhibit a distinct profile, characterized by broadened bases and sharp maxima. This indicates a convolution of overlapping reflections that suggest the coexistence of nanocrystalline/partially crystalline and well-crystallized phases. Indeed, T60 XRD pattern could not be well-fitted, applying only one magnetite phase. Therefore, two different cubic magnetite phases (Inorganic Crystal Structure Database CIF # 85806), referred to as M1 and M2, were used in the fitting. This is a result of a bimodal crystallite size distribution of magnetite in the samples. Although M1 and M2 are both magnetite, they presented different mean crystallite sizes and lattice parameters after the adjustment (Table 1). It can be observed that M1 has a smaller crystallite size and larger lattice parameter “*a”* than phase M2. M1 shows a very small crystallite mean size that reaches the detection limit of the XRD technique. For that reason, their mean sizes were determined as less than 5 nm instead of a value (Table 1). In addition to the M1 and M2 magnetite phases, samples T70 and T80 presented the hematite (ICSD CIF # 82902) phase. It can be observed in Figure 1 that the hematite characteristic peaks are more prominent in sample T80 than in sample T70. This is a result of the crystallite size growth from 5.4 to 20.0 nm with an increase of 10 °C in the synthesis temperature. It is difficult to affirm the quantity of hematite in T70 since the peaks are very broad with low intensity. It can be suggested that the formation of hematite starts to occur at this temperature. Magnetite is Fe_3_O_4_, a mixture of Fe^2+^ and Fe^3+^ oxides. The phase transformation from magnetite to hematite, Fe_2_O_3_, represents an oxidation of the Fe^2+^ of magnetite. The adjustment parameters of the Rietveld refinement method were GOF from 1.35 to 1.37 and *R*_wp_ from 1.45 to 1.54 for the three samples.

**Table 1:** Rietveld Refinement Results: Crystalline Phases Mass Percentages, Mean Crystallite Size, and Lattice Parameters^a^

	crystalline phases (% mass)			mean crystallite size (LVolIB/nm)			lattice parameters (Å)			
	M1	M2	H	M1	M2	H	M1	M2	H	
sample							a	a	a	c
T60	79.9	20.1		<5	9		8.4105	8.3664		
T70	78.6	13.4	8.0	<5	12	5	8.3850	8.3671	5.0567	13.7686
T80	75.9	15.4	8.7	<5	12	20	8.3789	8.3657	5.0517	13.7619

a M1 and M2 are magnetite phases, and H is the hematite phase; the mean crystallite sizes of the phases were obtained by the integral peak breadth-based volume calculation (LVollB).

Figures S1 and S2 (Supporting Information) show the contribution of each magnetite phase, M1 and M2, to the diffraction pattern of T80 that helps to understand the bimodal behavior. In addition, Figure S3 depicts the contribution of hematite in T80.

### 4.2 Raman Spectroscopy

The Raman spectra of the synthesized samples are displayed in Figure 2a, revealing three primary Raman bands at 341, 500, and 680 cm^–1^, characteristic of the presence of the magnetite phase.^19^ According to XRD analysis, hematite (α-Fe_2_O_3_) is present in the T70 and T80 samples at approximately 5 and 20 wt %, respectively, while no detectable hematite is observed in T60. However, only magnetite-related bands are visible in the Raman spectra of all three samples. The characteristic hematite peaks at 225, 295, and 410 cm^–1^ are not observed, even in T80, which contains the highest hematite content. This may be due to the higher Raman scattering efficiency of magnetite under the laser excitation used. Additionally, in multiphase systems, the broad and intense bands of magnetite could potentially overlap and obscure the weaker signals of hematite, particularly when hematite is present in relatively low amounts, as in T70 and even T80. Furthermore, it is also possible that the laser power used during Raman acquisition may induce partial phase transformation from hematite to magnetite, further contributing to the absence of detectable hematite signals.^20^ The position and full width at half-maximum (fwhm) of each peak were determined through peak deconvolution, and the results are presented in Figure 2b–d.

**Figure 2:**
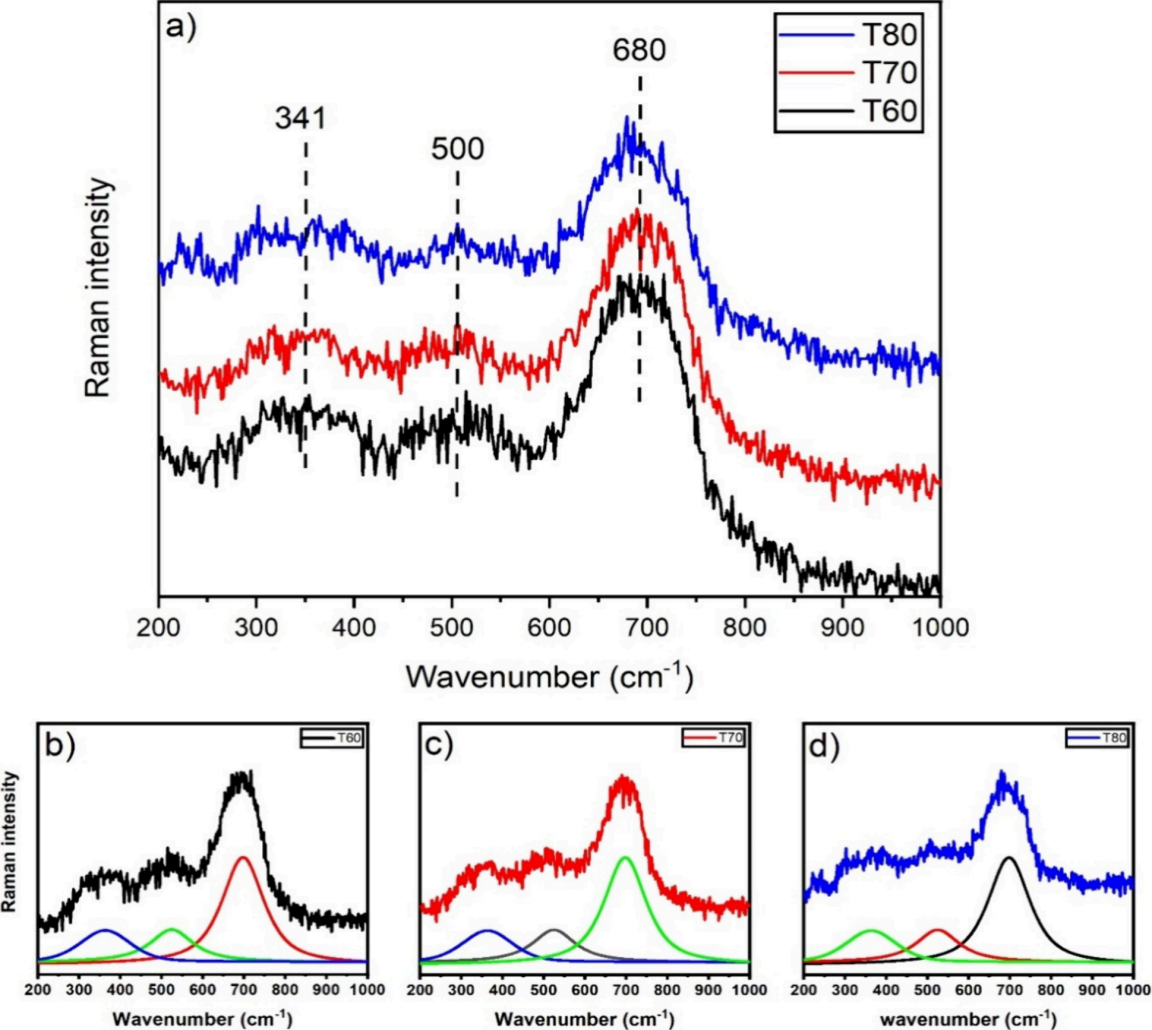
(a) Raman spectra of synthesized magnetite (Fe_3_O_4_) and peak deconvolution for the samples (b) T60 (black curve), (c) T70 (red curve), and (d) T80 (blue curve).

Raman spectroscopy was used to estimate the size of the synthesized Fe_3_O_4_ particles. eq 4 was used to infer the particle size from the Raman spectrum.

$$\Delta \omega =\omega (L)-\omega 0=A{\left(\frac{a}{L}\right)}^{\gamma }$$ 4

The frequency of the Raman peak is denoted as ω­(*L*), while ω0 represents the frequency of the Brillouin zone center. The difference between these frequencies is denoted as Δω. ‘*a*’ represents the crystal lattice constant, while ‘A’ and ‘γ’ are the fit parameters describing phonon confinement in nanocrystallites. The parameter ‘*a*’ is calculated using X-ray diffraction, and the parameters ‘A’ and ‘γ’ are constants obtained by Chandramohan.^21^ From eq 4, an inversion is performed, and eq 5 is used to estimate the particle size (*L*), as proposed by Chandramohan:^21^

$$L=a{\left(\frac{A}{\Delta \omega }\right)}^{1/\gamma }$$ 5

Using all this data, the particle sizes were calculated, and the results are shown in Table 2, along with the full width at half-maximum (fwhm) values.

**Table 2:** Particle Size Statistics from Peak 680 cm^–1^

sample	position (cm^–1^)	fwhm (cm^–1^)	size (nm)	standard error (nm)
T60	680.1	106.1	8.2	0.2
T70	680.7	99.6	8.9	0.2
T80	672.7	94.4	9.4	0.2

Since the Raman technique was not able to detect hematite, it can be assumed that the particle size obtained represents an average between the sizes of these two phases. Notably, this value is comparable to the mean crystallite size obtained by XRD, especially when considering the average hematite and magnetite values. This suggests that each particle may consist of a single crystallite.

### 4.3 Transmission Electron Microscopy

The three samples analyzed in this set of TEM micrographs have similar shapes, sizes, and modes of aggregation of nanoparticles (Figure 3a–c).

**Figure 3:**
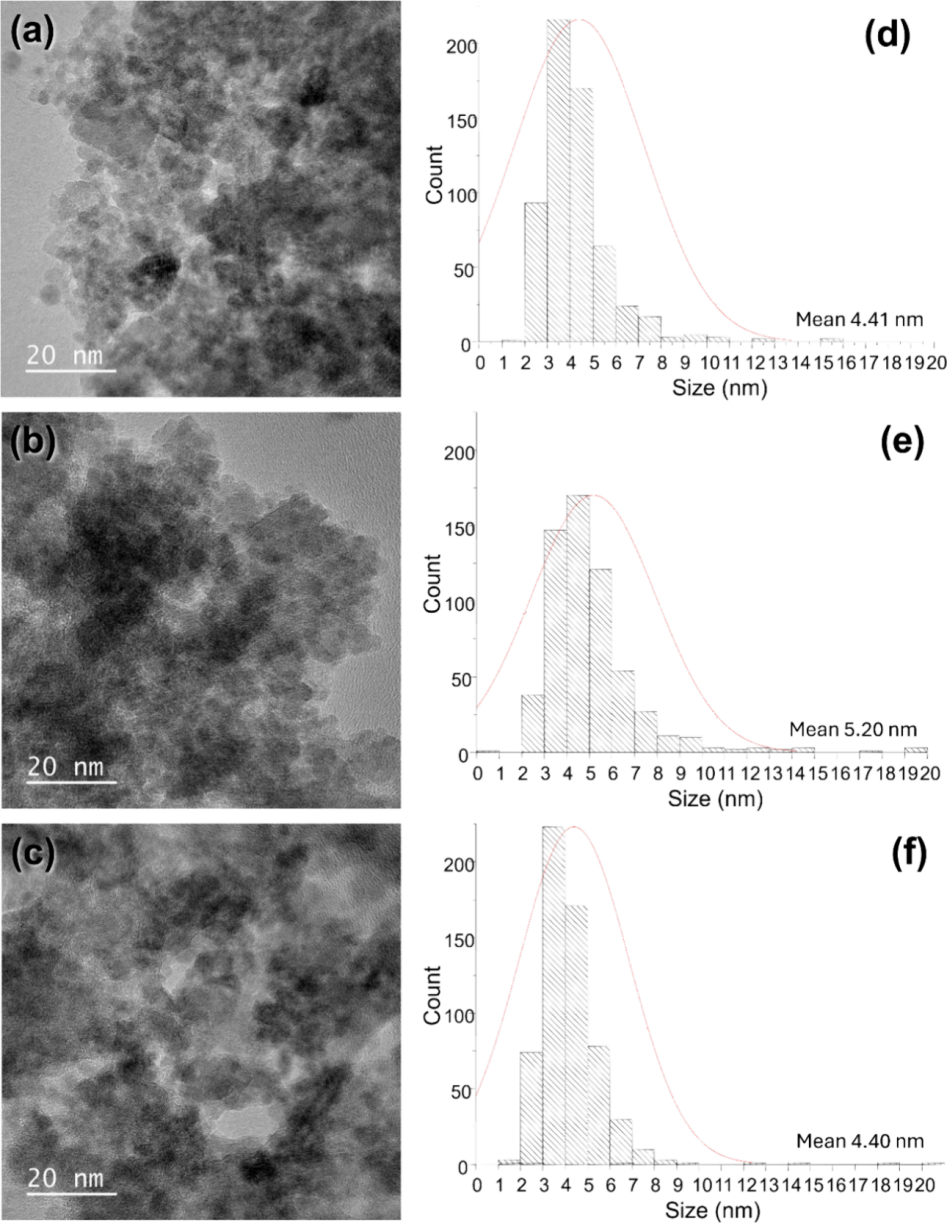
TEM images of the samples: (a) T60, (b) T70, and (c) T80. Histograms of the particle size of the samples: (d) T60, (e) T70, (f) T80. Population: 600 particles per sample.

The mean particle size and distribution were evaluated by measuring the diameter of six hundred particles of each sample using the free image analysis software “ImageJ”. The three samples displayed a similar size distribution. They had an average size of approximately 5 nm, with the presence of a small number of larger particles of size around 20–40 nm (faceted morphology). The statistical analysis of the population of measured particles (N) and mean size (nm) were obtained using the Origin software (Figure 3d–f). Compared to other synthesis methods found in the literature, our results indicate that most of the measured particles have a smaller average size. For instance, the sol–gel method typically produces particles in the range of 8–12 nm.^22,23^ By the microemulsion route at room temperature, they reported that uniform size and crystals with a spinel structure and average diameters of about 3, 6, and 9 nm were synthesized with high yield.^24^ Furthermore, Cabrera et al. reports that synthesis by the electrochemical route resulted in magnetite MNPs with sizes between 20 and 30 nm by electro-oxidation of Fe in the presence of an amine surfactant.^25^ These comparisons highlight the differences in particle size depending on the synthesis temperature, demonstrating the effectiveness of our method in achieving nanoscale control.

Table 3 shows that the mean diameter distribution results obtained using the TEM technique are consistent with those obtained using the XRD technique and Raman spectroscopy. All samples mostly present magnetic nanoparticles with diameters between 4 and 10 nm.

**Table 3:** Comparative Table of the Distribution of Mean Diameters Measured by the XRD, Raman, and TEM Techniques

	XRD (nm)				
sample	M1	M2	H	Raman (nm)	TEM (nm)
T60	<5	9.1		8.2 ± 0.2	4.4 ± 1.3
T70	<5	12.1	5.4	8.9 ± 0.2	5.2 ± 1.4
T80	<5	12.5	20	9.4 ± 0.2	4.4 ± 1.3

### 4.4 Electrical Measurements

In this work, an investigation was carried out to evaluate the correlation between DC electrical current density measurements and optical reflectance in samples T60, T70, and T80, aiming to associate electronic and optical properties and identify variations in the density of state occupancy within the valence and conduction bands as a function of synthesis temperature. The electrical response, particularly conductivity, directly influences the optical behavior by modulating reflectance through electronic transitions and charge carrier–photon interactions.^26^ In oxide-based materials such as magnetite, this correlation can be interpreted through classical free electron models, such as the Drude model.^27^

Figure 4a presents the reflectance spectra as a function of photon energy. In the visible region (highlighted by the shaded area), a general decrease in reflectance is observed with increasing energy. Notably, the reflectance is lower for the sample synthesized at 80 °C across the entire visible energy range, compared to T60 and T70. Figure 4b displays the current density as a logarithmic function of applied voltage (ln­(*J*)–*V*). The data reveal an increase in current density with applied voltage for all samples, with the T80 sample showing a significantly higher current density, especially beyond 5 V, when compared to T60 and T70. This indicates enhanced electrical conduction at higher synthesis temperatures. Figure 4c complements the electrical characterization by presenting the *I*–*V* characteristics, where the same trend is evident: samples synthesized at higher temperatures exhibit increased current flow under the same applied voltage conditions, reinforcing the conductivity enhancement observed in Figure 4b.

**Figure 4:**
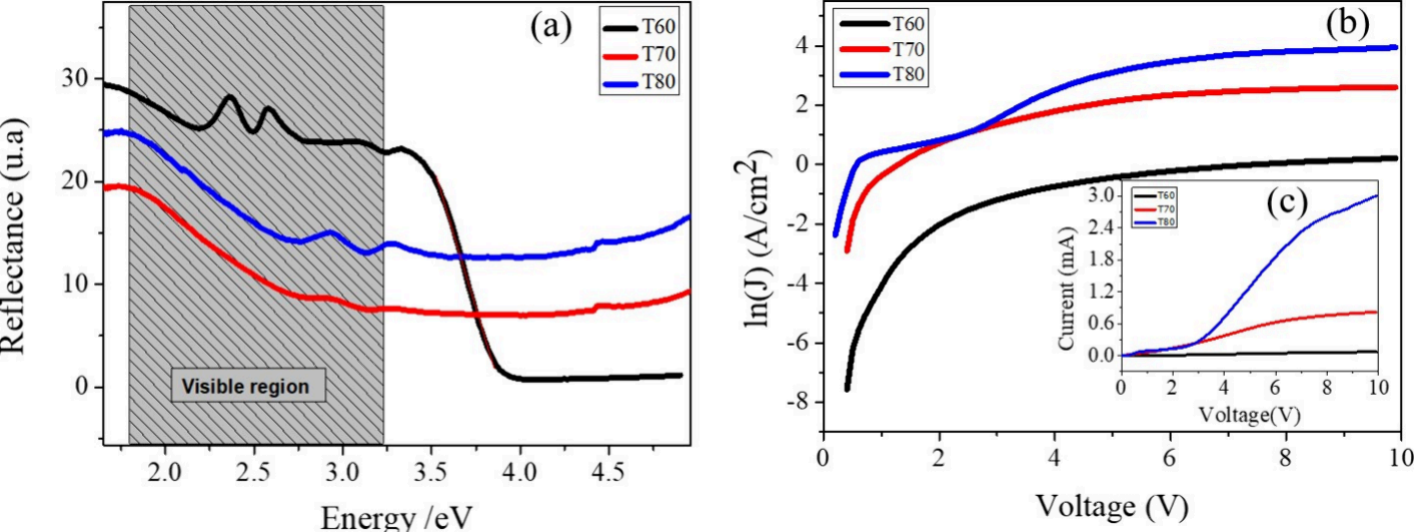
Analysis of samples T60, T70, and T80. (a) Reflectance spectra as a function of photon energy, highlighting the visible region. (b) Current density as a logarithmic function of voltage (ln­(*J*)–*V*). (c) Current versus voltage (*I*–*V*) characteristics for each sample.

From Figure 4a–c, it is evident that both optical reflectance and electrical conductivity are strongly influenced by the synthesis temperature. As temperature increases, reflectance decreases while current density and current increase. The higher reflectance observed in the T60 sample implies lower light absorption and suggests a reduced density of free electronic states available for conduction. In contrast, the lower reflectance and higher current response in T80 indicate a greater density of electronic states and enhanced charge carrier mobility. These observations confirm that synthesis temperature significantly alters the electronic structure and transport properties of the material temperature.^28^

### 4.5 Reflectance

It is known that temperature can directly influence the optical properties of a material, being possible to change its refractive index,^29,30^ absorption,^31^ and light scattering.^32^ In all samples (Figure 5), the peaks at 478 and 522 nm can be noticed because the samples are composed of Fe_3_O_4_.

**Figure 5:**
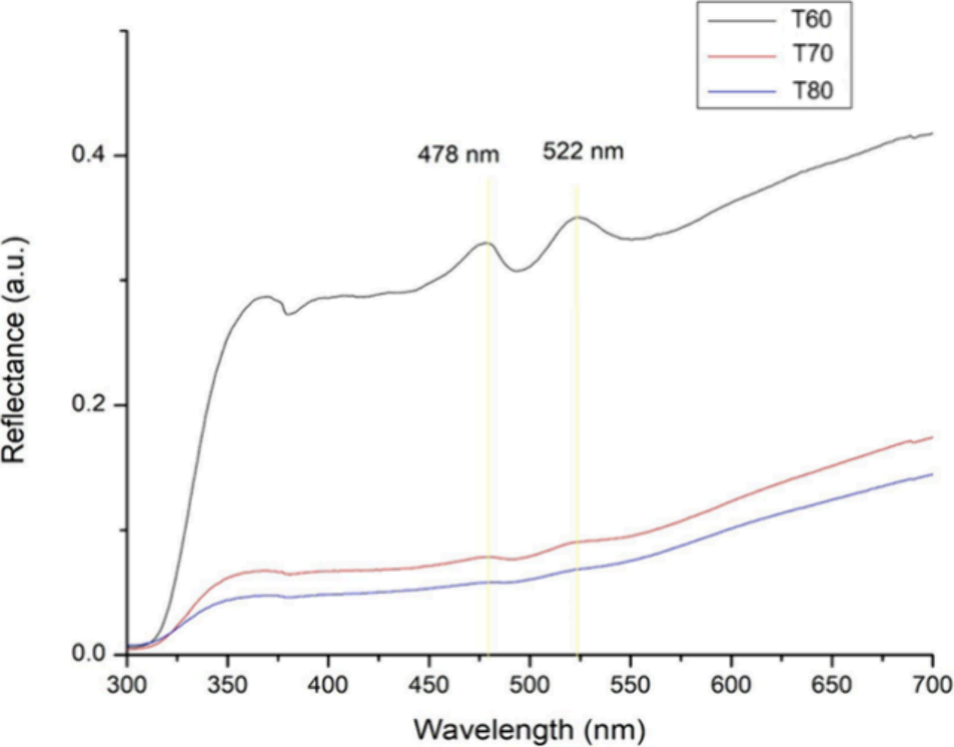
Reflectance spectrum of magnetic nanoparticle samples obtained by the UV–vis–NIR spectrophotometer.

Around 350 nm, there is a change from a deuterium lamp to a tungsten-halogen lamp, as observed through the jump in the reflectance around this value. Despite this, it is possible to observe a decrease in reflectance between the samples, as they were produced in different ways. In each sample, the precipitating agent has a different temperature, and this change in synthesis generates a more defined reflectance spectrum. There is an inversely proportional relationship: the lower the temperature of the precipitating agent, the greater the reflectance presented by the material; the higher the temperature of the precipitating agent, the lower the reflectance presented by the material.

### 4.6 Magnetic Measurements

Figure 6 shows the magnetization versus applied field curves for the investigated samples measured in the range of ±20 kOe at room temperature. With increasing synthesis temperature, a slight decrease in magnetization is observed. At the maximum applied field (20 kOe), the magnetization of samples T70 and T80 decreased by 3.9 and 7.4%, respectively, compared to T60. This behavior is directly related to the relative increase in the hematite phase, as evidenced by the XRD results. The hematite phase exhibits lower magnetization compared to magnetite due to its corundum-type crystalline structure being antiferromagnetic, where partial magnetic moments cancel each other, resulting in a significantly lower net magnetization compared to the inverse spinel structure of magnetite, which allows significant contributions from its magnetic sublattices.^33^ Although the coercive fields are very low, on the order of 25 Oe, their values exceed the magnetometer’s remanent field (5–10 Oe), confirming the instrumental contribution is not the dominant factor.^34,35^

**Figure 6:**
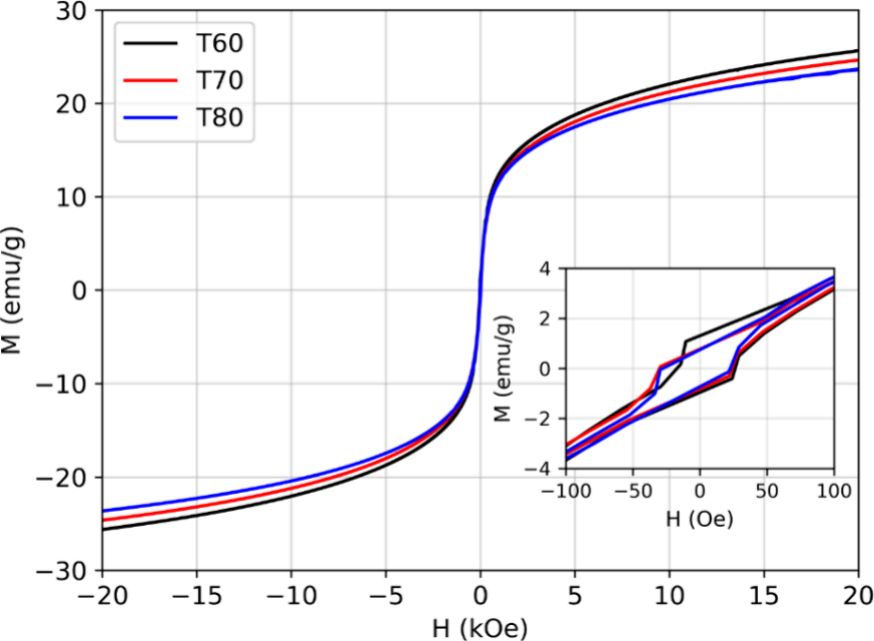
Magnetization vs applied field curves of the samples T60, T70, and T80. The inset shows the magnetization in the low-field region (−100 to 100 Oe).

The temperature dependences of magnetization were recorded in zero-field-cooled (ZFC) and field-cooled (FC) modes under an applied field of 100 Oe for the samples obtained at different synthesis temperatures. These measurements were conducted over a temperature range of 2–400 K to investigate the thermal behavior and magnetic transitions of the samples. The results are presented in Figure 7. The average blocking temperatures (*T*_B_), determined from the peak in the ZFC curves, were approximately 180 K, while the irreversible temperatures (*T*_IRR_), indicated by the bifurcation between ZFC and FC curves, were around 370 K for T60, 360 K for T70, and 375 K for T80. Table 4 summarizes the magnetic parameters derived from our magnetization measurements. While this variation in *T*_B_ and *T*_IRR_ could suggest differences in particle size distribution, our results (XRD, RAMAN, and TEM) confirm a relatively narrow size distribution, implying that other factors may also contribute to the observed thermal behavior.

**Figure 7:**
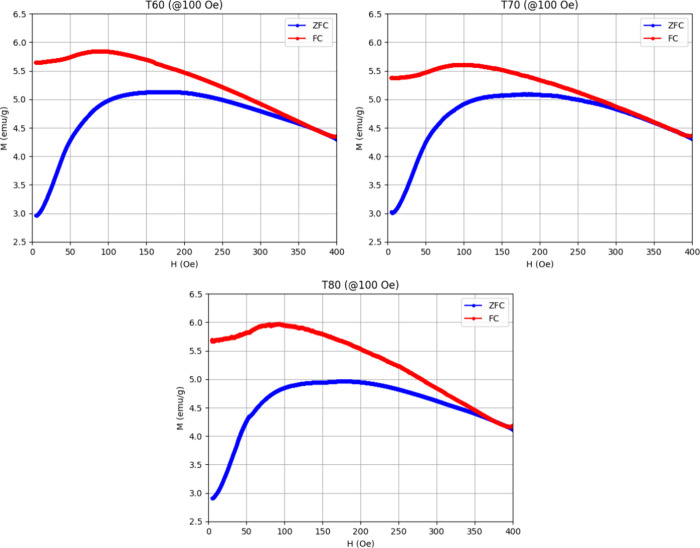
Magnetization vs temperature curves for the samples T60, T70, and T80, measured in ZFC and FC modes under an applied field of 100 Oe.

**Table 4:** Summary of Magnetic Characterization Results

sample	*M* at 20 kOe (emu/g)	*M*_r_ (emu/g)	*H*_c_ (Oe)	*T*_B_ (K)	*T*_IRR_ (K)
T60	25.6	1.13	21.5	177	370
T70	24.6	0.80	27	179	360
T80	23.7	0.74	25.2	181	375

Dipolar and exchange interactions between nanoparticles, arising from the presence of agglomerates or larger particles, can influence *T*_B_ and *T*_IRR_, potentially masking the direct effect of the increasing hematite phase and further complicating the interpretation of the thermal response.^36^ These combined effects suggest that the observed trends in *M*(*T*) are not solely governed by particle size variations but also by interparticle interactions and structural modifications induced by the synthesis conditions. Indeed, our measurements reveal strong interparticle magnetic coupling, as evidenced by three key features: (1) a pronounced gap between the ZFC and FC curves (difference between *T*_IRR_ and *T*_B_ ≈ 190 K), (2) significant broadening of ZFC curves, and (3) a flattened slope in the FC curve, along with the emergence of a peak, both occurring below *T*_B_.^37–39^ Taken together, these signatures point to dominant dipolar and/or surface-mediated interactions, consistent with the magnetic behavior typically observed in densely packed or strongly interacting nanoparticle systems.

## 5 Conclusions

The results obtained by the characterization techniques (XRD, Raman, and TEM) confirmed the average diameter size of the nanoparticles. They indicated that the synthesis temperature in the range of 60 to 80 °C did not significantly affect the average size of the nanoparticles. XRD analysis revealed the characteristic peaks of magnetite for samples T60, T70 and T80. Using the Topas 5.0 program, it was possible to adjust two different cubic magnetite phases, i.e., different mean crystallite size and lattice parameters; XRD also detected the hematite phase in samples T70 and T80. The broadening of the peaks in the diffractogram suggests the formation of nanometer-scale particles. Raman spectroscopy showed the characteristic bands of the magnetite phase. This method also provided an estimate of the average diameter size of magnetic nanoparticles, which was 8.2 ± 0.2, 8.9 ± 0.2, and 9.4 ± 0.2 nm for samples T60, T70, and T80, respectively. TEM imaging showed that most of the samples had cubic-shaped particles with average sizes of 4.4, 5.2, and 4.4 nm for samples T60, T70, and T80, respectively. Electrical measurements showed that synthesis above 60 °C alters the material’s energy band, which can lead to a higher density of free electronic states. This, in turn, can boost the samples’ electrical conductivity, making them more effective for use in electronic and optoelectronic devices.^40^ Magnetic characterization revealed that increasing synthesis temperature leads to a decrease in magnetization due to the formation of the hematite phase. Additionally, dipolar and exchange interactions may influence the thermal response, suggesting that interparticle effects contribute to the observed trends in magnetization. Due to their size and magnetic characteristics, MNPs obtained by this synthesis route demonstrate potential for medical/biological applications, image analysis and drug nanocarriers. T80 is a promising material for adsorption of contaminants since its increased percentage of hematite may result in enhanced catalytic and magnetic properties. The materials produced show also good thermal and chemical stability.

## Supplementary Material



## Notes

Contribution of each magnetite phase, M1 and M2, to the diffraction pattern of T80 that helps understand the bimodal behavior; and contribution of hematite in T80 (PDF)
