# Supplementary material for: Tuning Magnetic Nanoparticles: Effect of Temperature on the Formation of Magnetite and Hematite Phases
Source: ACS Omega. 2025 Jun 11;10(24):26023–32. doi: 10.1021/acsomega.5c02750 (PMC12199064; doi:10.1021/acsomega.5c02750)
Supplement: Supplementary file 1 [file ao5c02750_si_001.pdf]

Tuning Magnetic Nanoparticles: Effect of Temperature on the Formation of Magnetite and Hematite Phases

Frederico Vieira Gutierrez[1*], Sonia Letichevsky[2,3], Anna De Falco[4,5], Beatriz Marques Ereias[1], Caique Diego de Abreu Lima[1], Wanessa Afonso de Andrade[1], Lanna Isabely Morais Sinimbu[1], Thais Cristina Viana de Carvalho[1,6], Bruno Gomes Silva[7], Rubem Luis Sommer[7], Geronimo Perez[8], Liying Liu[7] and Jefferson Ferraz Damasceno Felix Araujo[1**]

[1] *Department of Physics, Pontifical Catholic University of Rio de Janeiro, Rua Marques de São Vicente, 225, 22451-900, Rio de Janeiro, Brazil*
[2] *Department of Chemical and Materials Engineering, Pontifical Catholic University of Rio de Janeiro, Rua Marques de São Vicente, 225, 22451-900, Rio de Janeiro, Brazil*
[3] *New Wave Tech, Av. Mascarenhas de Morais, 2231, 25230-030, Rio de Janeiro, Brazil*
[4] *Center for Biotechnology and Interdisciplinary Sciences, Rensselaer Polytechnic Institute, Troy, NY 12180, USA*
[5] *Department of Chemistry and Chemical Biology, Rensselaer Polytechnic Institute, Troy, 12180, NY, USA*
[6] *NIT – National Institute of Technology, Venezuela Avenue, 82, 20081-312, Rio de Janeiro – RJ, Brazil*
[7] *Brazilian Center for Research in Physics, 22290-180, Rio de Janeiro, RJ, Brazil*
[8] *Department of Mechanical Engineering, Universidade Federal Fluminense, 24210-240, Niteroi, RJ, Brazil*

[*]Corresponding author.
[**]Corresponding author.
E-mail: frederico.vgutierrez@gmail.com
E-mail: jferraz@puc-rio.br

Supplementary material

Figures S1 and S2 show the contribution of each magnetite phase, M1 and M2, to the diffraction pattern of T80 that helps to understand the bimodal behavior. In addition, Figure S3 depicts the contribution of hematite in T80.

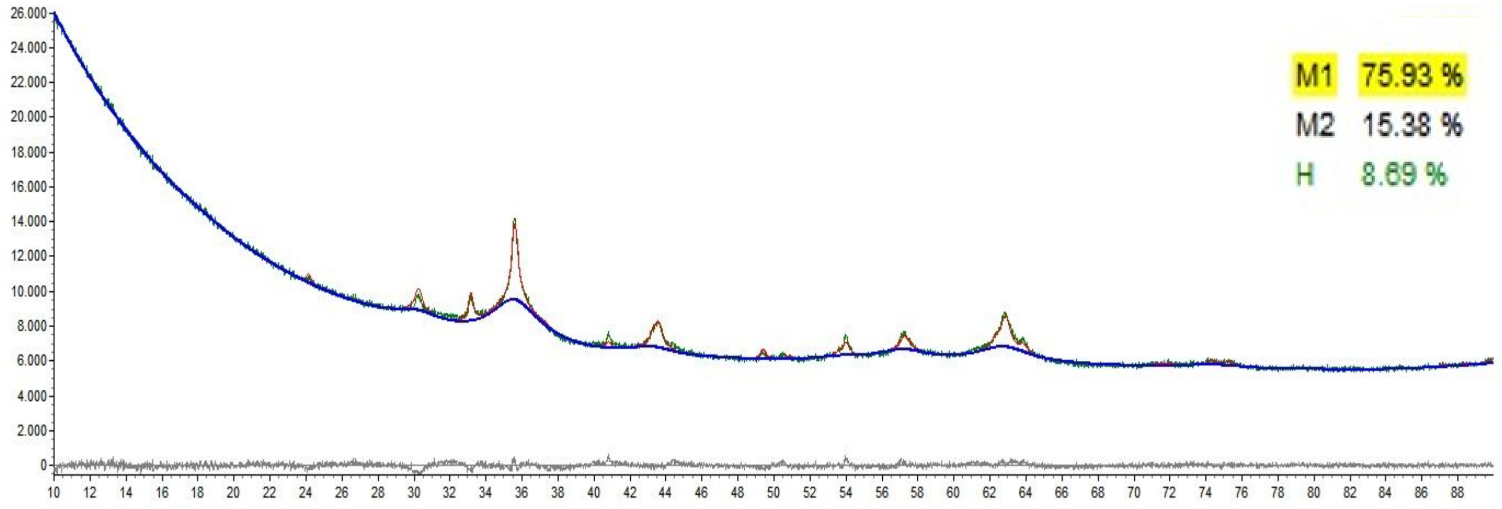

Figure S1. T80 diffraction pattern showing the contribution of M1 to the peaks (blue).

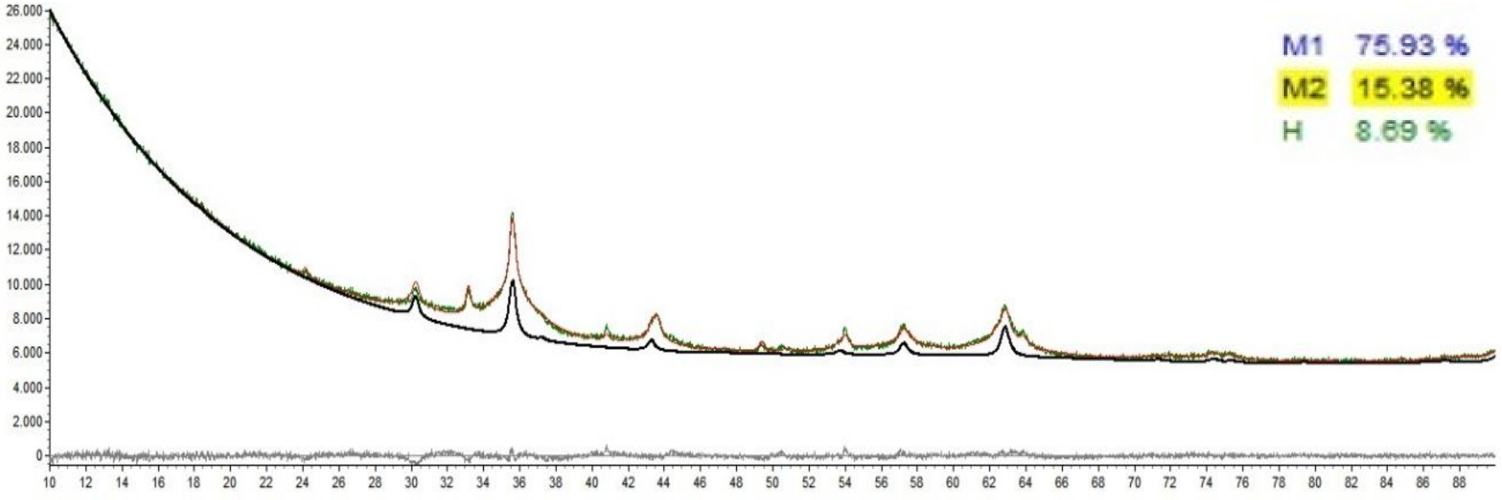

Figure S2. T80 diffraction pattern showing the contribution of M2 to the peaks (black).

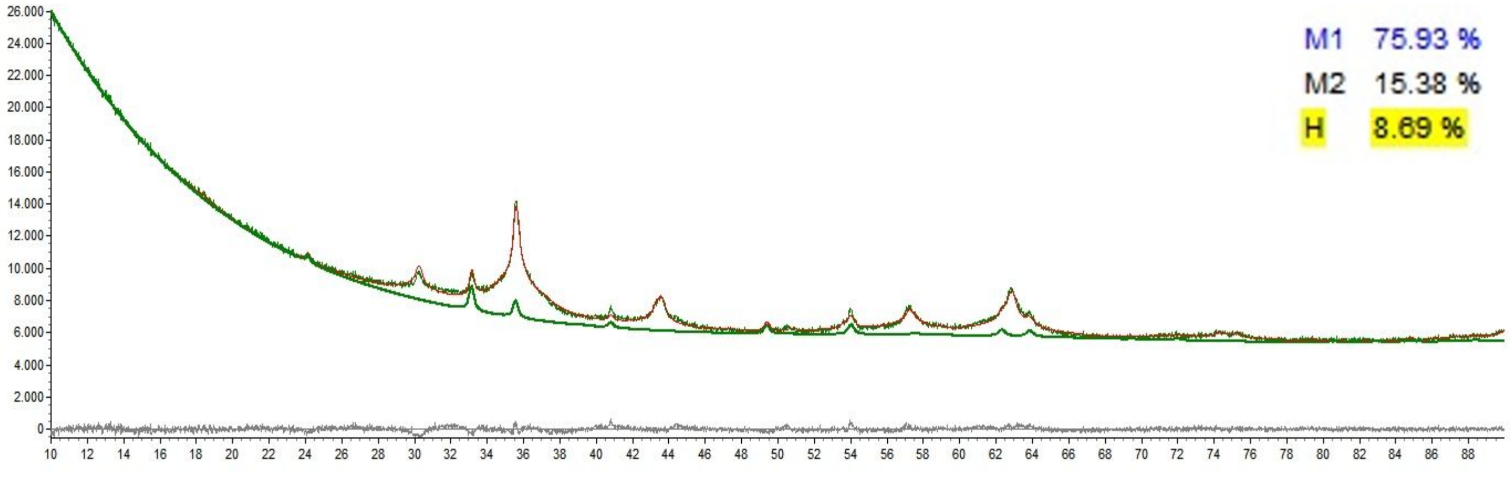

Figure S3. T80 diffraction pattern showing the contribution of M2 to the peaks (green).
